# The different roles of cyclinD1-CDK4 in STP and mGluR-LTD during the postnatal development in mice hippocampus area CA1

**DOI:** 10.1186/1471-213X-7-57

**Published:** 2007-05-30

**Authors:** Chenchen Li, Xinmei Li, Weiheng Chen, Shanshan Yu, Jutao Chen, Huili Wang, Diyun Ruan

**Affiliations:** 1 Department of Neurobiology and Biophysics, School of Life Sciences, University of Science and Technology of China, Hefei, Anhui 230027, P.R. China

## Abstract

**Background:**

Cell-cycle-related proteins, such as cyclins or cyclin-dependent kinases, may have functions beyond that of cell cycle regulation. The expression and translocation of cyclinD1-CDK4 in post-mitotic neurons indicate that they may have supplementary functions in differentiated neurons that might be associated with neuronal plasticity.

**Results:**

In the present study, our findings showed that the expression of CDK4 was localized mostly in nuclei and cytoplasm of pyramidal cells of CA1 at postnatal day 10 (P10); whereas at P28 staining of CDK4 could be detected predominantly in the cytoplasm but not nuclei. Basal synaptic transmission was normal in the presence of CDK4 inhibitor. Short-term synaptic plasticity (STP) was impaired in CDK4 inhibitor pre-treated slices both from neonatal (P8-15) and adolescent (P21-35) animals; however there was no significant change in paired-pulse facilitation (PPF) in slices pre-incubated with the CDK4 inhibitor from adolescent animals. By the treatment of CDK4 inhibitor, the induction or the maintenance of Long-term potentiation (LTP) in response to a strong tetanus and NMDA receptor-dependent long-term depression (LTD) were normal in hippocampus. However, long-term depression (LTD) induced either by group I metabotropic glutamate receptors (mGluRs) agonist or by paired-pulse low-frequency stimulation (PP-LFS) was impaired in CDK4 inhibitor pretreated slices both from neonatal and adolescent animals. But the effects of the CDK4 inhibitor at slices from adolescent animals were not as robust as at slices from neonatal animals.

**Conclusion:**

Our results indicated that the activation of cyclinD1-CDK4 is required for short-term synaptic plasticity and mGluR-dependent LTD, and suggested that this cyclin-dependent kinase may have different roles during the postnatal development in mice hippocampus area CA1.

## Background

Cyclin D1, a member of the G1 cyclins, plays an important role in the G1 phase progression of the cell cycle in proliferating cells via activation of cyclin-dependent kinase 2 (CDK2), CDK4, or CDK6. The cyclinD/CDK4/6 complexes induce the phosphorylation of retinoblastoma (Rb) protein and the release of E2F, which trigger G1 cell cycle progression. Normally, Rb binds to the members of the E2F family of transcription factors [1]. The expression and location of cyclin D1 and CDK4 in G1 cell cycle acts as the primary sensors of positive and negative environmental signals [2-4]. Studies report that the expression and translocation of cyclinD1-CDK4 are regulated by several signaling molecules, including PI3K/AKT/mTOR/p70S6K1 signaling [5-8], JNK [9], Rho [10] and NF-kappaB [11]. All of them are present in the hippocampus, where they participate in the regulation of synaptic functionality and gene transcription [12-14]. However, the expression of cell cycle markers in the postnatal or adult brain is still a matter of controversial debate. Ectopically expressed cyclinD1 sequestered in the cytoplasm of post-mitotic neurons, whereas it efficiently entered the nucleus of proliferating progenitor cells [15-17]. Even in the adult mouse hippocampus, cyclinD1-CDK4 were found in terminally differentiated pyramidal and granule neurons, and also were found in dendrites and mossy fibers[18].

Paradoxically, increasing evidence suggests that, cell cycle arrest and terminal differentiation of neurons may not be necessarily incompatible with CDK activity but raise the possibility that CDKs and cyclins have physiological impact general effects, such as metabolic regulation [19], basal transcription [20,21], apoptosis [22,23] or mechanisms of neuronal and synaptic plasticity [24]. A further function might be implicated in regulating microtubules stability [25] thereby influencing morphoplastic processes. The present results suggest that the expression of cell cycle-related markers may have supplementary functions in differentiated neurons that might be associated with neuronal plasticity. In this study, we initiated to investigate the potential functions of cyclinD1-CDK4 in neuronal plasticity during the postnatal development in mice hippocampus area CA1.

## Results

### Neuronal expression and sub-cellular localization of cyclinD1-CDK4 during the postnatal development in hippocampus area CA1

**Figure 1:**
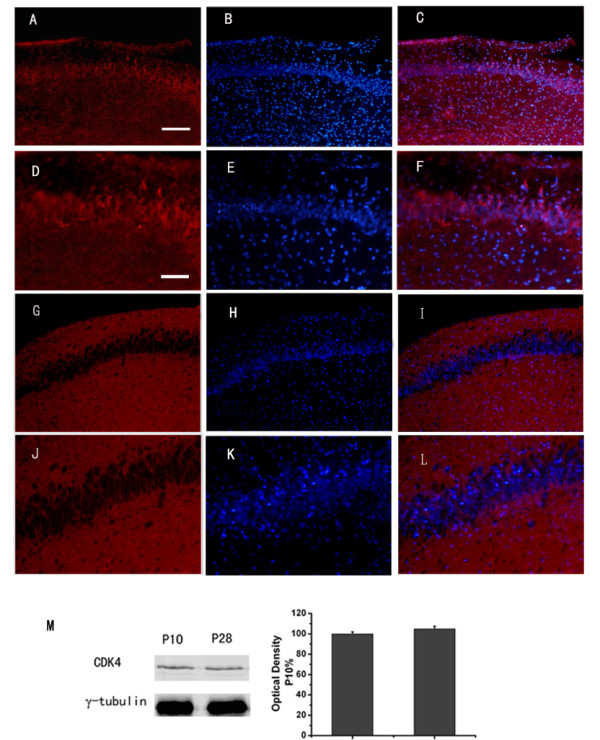
**Expression and localization of cyclinD1-CDK4 during the postnatal development in hippocampus area CA1**. Immunofluorescent microscopic localization of CDK4 in hippocampus area CA1 at PND10 (A-F) and at PND28 (G-L). Blue indicates nuclear DAPI counterstain, and red indicates CDK4. C, F, I, L are the merged image. Scale bar: in *A *(applies to *A*-C and G-I), 121 μm; in *D *(applies to *D-F *and *J-L*), 48 μm. Detection of CDK4 on Western blots (M). PND (postnatal day)

At P10, hippocampal genesis is almost completed and the typical lamination is finished. Perikarya of the stratum pyramidal display the characteristic pyramidal shape [18]. The immunofluorescent detection of CDK4 demonstrated a neuronal expression at P10 (Fig. 1A–F). Expression of CDK4 was localized both in nuclei and cytoplasm of pyramidal cells of CA1 regions. In addition, mossy fibers were immuno-reactive as well (data not shown). At P28. The sub-cellular localization of CDK4 was different. Staining of CDK4 could be detected predominantly in the cytoplasm but only sporadically in neuronal nuclei of pyramidal neurons of the CA subfields (Fig. 1G–L). Further quantitative immunoblotting analysis confirmed the similar expression levels of CDK4 in P10 and P28 mice hippocampus (Fig. 1M).

### Basal synaptic transmission was normal in the presence of CDK4 inhibitor

**Figure 2:**
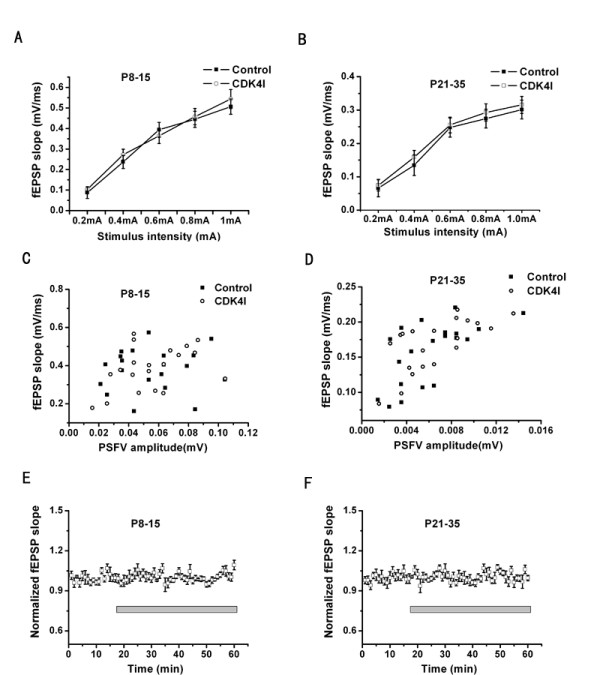
**Basal synaptic transmission is normal in the presence of CDK4 inhibitor**. (A, B): Incubation in 5 μM CDK4 inhibitor(CDK4I) (n = 16 slices) for 30 min before recording had no effect on stimulus-response curves of fEPSP slope (mV/ms) vs. stimulus (mA) at the SC-CA1 synapses in hippocampal slices both from P8-15 and P21-35 mice(control, n= 12slices). Data are presented as mean ± standard error (SE). (C, D): plots of fEPSP slope (mV/ms) vs. presynaptic fiber volley amplitude (mV) from a random sample of slices from P8-15 and P21-35 animals. No differences were apparent between control (*n *= 20 slices) and CDK4I pretreated slices (*n *= 20 slices). (E, F)slices (*n *= 16 slices) were stimulated at a stimulus intensity that produced 50% of the maximal fEPSP for 15 min.CDK4I was then introduced into the perfusion buffer [artificial cerebrospinal fluid (ACSF)] at a concentration of 5 μM and responses were recorded for 1 h. No change in the fEPSP was observed both from P8-15 and P21-35 mice. Error bars correspond to SE.

Having localized CDK4 in the hippocampus, we next determined the role of cyclinD1-CDK4 in synaptic plasticity. We first examined whether the complex were required for normal basal synaptic transmission. We compared extracellular recordings of the SC-CA1 synapses in hippocampal slices (n = 12 slices from 4 animals) to slices pretreated with CDK4 inhibitor (n = 16 slices from 5 animals). There was not significantly different both at neonatal and adolescent animals (P = 0.897 and 0.826 respectively, analysis of variance (ANOVA); Fig. 2A, B). Furthermore, the fEPSP slope corre-sponding to a given presynaptic fiber volley was also unaffected by CDK4 inhibitor application throughout development (P = 0.883 and 0.437, respectively, analysis of variance (ANOVA); Fig. 2C, D). Bath application of 5 μM CDK4 inhibitor to slices from P8-15 and P21-35 animals did not affect the fEPSP slope, even after 1 h in the perfusion buffer (*n *= 16 slices from 5mice; Fig. 2E, F). Previous studies have shown that these concentrations are adequate to inhibit CDK4 kinase activity in cells [26].

### CyclinD1-CDK4 mediated short-term synaptic plasticity

**Figure 3:**
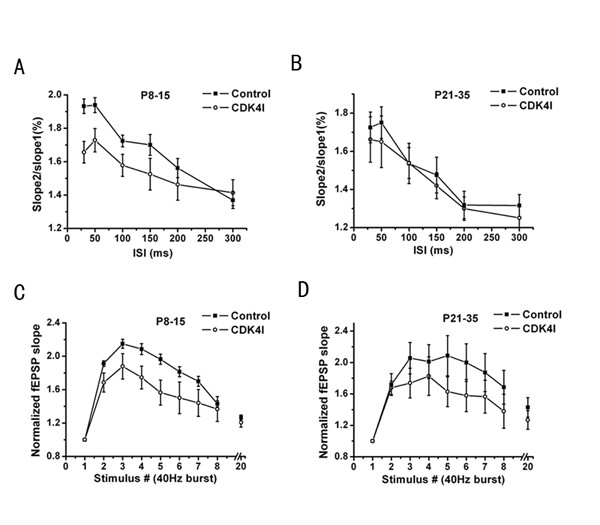
**Role of cyclinD1-CDK4 in short-term synaptic plasticity**. (A) Comparison of paired-pulse facilitation (PPF) in controls (n = 10 slices) and CDK4 inhibitor (5 μM) pre-treated slices (n = 11 slices). PPF ratio (slope2/slope1) measured at different interstimulus intervals (ISI) was significantly decreased in slices treated CDK4 inhibitor, at the neonatal stage (P8-15) (P < 0.01). (B) There was no significant decrease in PPF when applied CDK4 inhibitor to slices from adolescent animals (P21-35) (n = 12 slices). Data are presented as mean ± standard error (SE). (C, D) Frequency-dependent facilitation and posttetanic potentiation (PTP) in control and CDK4 inhibitor pre-incubated slices. Ratio of responses compared with the slope of the first fEPSP of a short stimulus train (8 stimuli of 50 μs duration at 40 Hz), followed by a single test stimulus delivered 300 ms after the burst. Responses of CDK4 inhibitor pretreated (*n = *12 slices and 11 slices) slices were significantly inhibited compared with control slices (*n = *11 slices and 10 slices) at both P8-15 and P21-35 (P < 0.01 and 0.005, respectively). Data are presented as mean ± standard error (SE).

We next examined whether the complex were involved in paired-pulse facilitation, a form of short-term plasticity. As shown in Fig. 3, at six different inter-stimulus intervals ranging from 30 to 300 ms, the mean paired-pulse facilitation ratio (second fEPSP slope/first fEPSP slope) was reduced in CDK4 inhibitor pre-incubated slices (n = 11 slices from 3 mice) as compared with control (n = 10 slices from 3 mice), at time intervals from 30 to 200 ms (p < 0.01, Fig. 3A), at the neonatal stage (P8-15), surprisingly there was no significant decrease in PPF when applied to slices from adolescent animals (P21-35) (n = 12 slices from 4 mice respectively; P = 0.217, Fig. 3B). These results demonstrated that cyclinD1-CDK4 may have the effect of modulating pre-synaptic function at the neonatal stage but not at the adolescent stage. This difference may be the consequence of the expression and translocation of cyclinD1-CDK4 in neuronal cells in area CA1 at different development stage [18].

As a second measure of short-term synaptic plasticity, we next compared the time evolution of the fEPSP slopes during and following a brief tetanic stimulation. The Schaffer collaterals were stimulated by a short 40 Hz train (8 stimuli) followed by a test stimulus delivered 300 ms after the end of the burst. We found that in the CDK4 inhibitor pretreated slices facilitation was greatly reduced during the burst (stimuli 2, 3, 4, 5, 6, 7)(n = 12, 11 slices from 4 mice for P8-15, P21-35; P < 0.01 and 0.005, respectively; Fig. 3C and 3D) compared with control slices (n = 11, 10 slices from 3mice for P8-15, P21-35, respectively) and while the 300 ms after the burst was not significantly different (ANOVA, P = 0. 86 and 0.37 compared with control, respectively). These observations suggested that cyclinD1-CDK4 may regulate the availability of vesicles from the readily releasable pool during repetitive stimulation during the postnatal development.

### CyclinD1-CDK4 had no effect on the induction or the maintenance of Long-term potentiation in the hippocampal CA1 region

**Figure 4:**
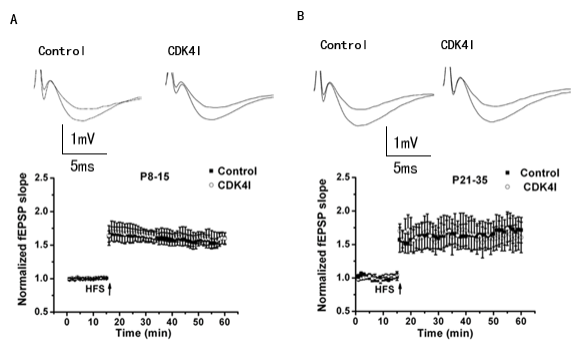
**There was no influence of CDK4 inhibitor on the induction or the maintenance of long-term potentiation in the hippocampal CA1 region**. LTP was induced by HFS (100 Hz, 1s). No significant difference was observed between LTP in slices from control (n = 13 slices) and CDK4 inhibitor pretreated slices (n = 11 slices) both at P8-15 (A) and P21-35 (B). Values were presented as a percentage of baseline (mean ± SEM).

To examine the role of cylinD1-CDK4 in long-term synaptic plasticity, we tested LTP at the SC-CA1 synapses. LTP was induced by HFS (100-Hz, 1s) to the Schaffer collateral inputs. The amount of LTP was quantified as the average fEPSP slope 45 min after HFS relative to the baseline mean slope. Inhibition of CDK4 had no significant effect on the induction or maintenance of LTP after 45 min both for P8-15 (control 163 ± 3%, 13 slices from 5 mice; control + CDK4I 164 ± 6%, 11 slices from 5 mice; Fig. 4A) and P21-35 (control 162 ± 8%, 13 slices from 6mice; control + CDK4I 167 ± 6%, 11 slices from 5mice; Fig. 4B). We did not examine whether the expression of this form of LTP or other forms of LTP, such as those induced by theta stimulation or non-NMDA receptor-dependent LTP in the CA3 region, are equally unaffected by CDK4 inhibitor.

### CyclinD1-CDK4 were required for mGluR-dependent LTD in the hippocampal CA1 region

**Figure 5:**
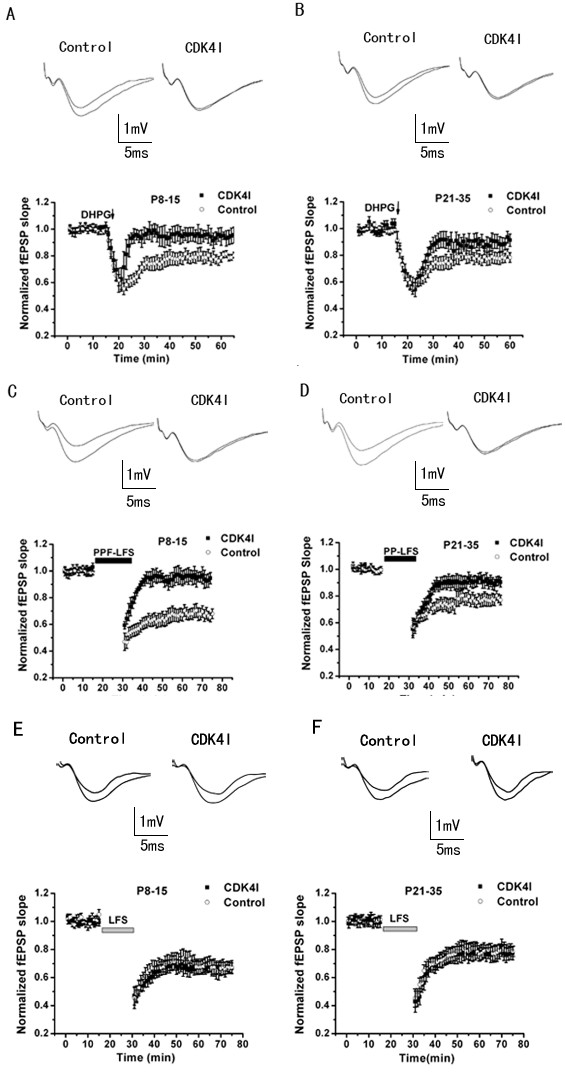
**CDK4 inhibitor impairs mGlu-LTD in the hippocampal CA1 region. **(A, B) DHPG-induced LTD. Slices were treated with 100 μM DHPG for 5 min (arrow). The effect is prominent in control slices (n = 11 slices), but impaired in CDK4 inhibitor reincubated slices (n = 12 slices) at both P8-15 and P21-35. The effects of the CDK4 inhibitor at slices from adolescent animals were not as robust as slices from neonatal animals on this form of LTD. (C, D) PP-LFS-induced LTD. The protocol consisted in pairs of stimuli with a 50 ms interstimulus interval, delivered at 1Hz for 15 min (1800 pulses total) in the presence of DL-AP-5 (100 μM). CDK4 inhibitor also inhibited this form of LTD. (E, F) Pooled data show that CDK4 was not required for NMDA receptor-dependent LTD induced by LFS (900 pulses at 1 Hz).

A metabotropic glutamate receptor-dependent form of LTD (mGluR-LTD) is expressed more strongly in neonatal synapses and can be preferentially induced by DHPG, a selective group I mGluR agonist. Therefore, the ability to develop and maintain this form of LTD was tested by applying DHPG (100 μM) to hippocampal slices. As shown in Fig. 4, CDK4 inhibitor did not alter the acute depression of synaptic transmission induced by DHPG, in contrast, 10 min after washout of DHPG, the fEPSP slope increased gradually in CDK4 inhibitor pretreated slices, while in control slices it remained depressed. In control slices, the fEPSP slope was reduced to 79 ± 2.8% of the baseline average value 45 min after application of DHPG (n = 15 slices from 5 mice), whereas, preincubation of slices with the CDK4 inhibitor, significantly inhibited the later phase of DHPG-LTD, fEPSP slope had recovered to 95 ± 4.1% of average baseline (n = 12 slices from 6 mice; ANOVA, p < 0.001) (Fig. 5A) in neonatal animals (P8 -P15). DHPG-LTD was also reduced by CDK4 inhibitor in adolescent animals (P21-P35) compared with control slices (DHPG plus vehicle, 78 ± 3% of the baseline, n = 15 slices from 5 mice; DHPG plus CDK4 inhibitor, 89 ± 3.6%, n = 12 slices from 6 mice, ANOVA, p < 0.01) (Fig. 5B).

When LTD was induced by PP-LFS (paired pulse separated by 50 ms were delivered at low frequency 1 Hz for 15 min in the presence of the NMDAR antagonist D-AP-5), the results on LTD induction and maintenance were similar to those obtained with DHPG-induced LTD. The later phase of the PP-LFS-induced LTD almost disappeared by CDK4 inhibitor (fEPSP slope returned to 96 ± 4%, n = 12 slices from 5 mice) in the P8-15 group while the fEPSP slope in the P21-35 group (n= 11 slices from 5mice) recovered to 91 ± 3.2% of the baseline at 45 min (ANOVA, p < 0.001 and 0.01, respectively) (Fig. 5C and 5D).

A different form of LTD, NMDA receptor dependent LTD (NMDAR-LTD), has been described at CA1 synapses. Consistent with the idea that mGluR- and NMDAR-LTD are mediated by distinct cellular mechanisms [27,28], CDK4 inhibitor- treated slices showed similar levels of NMDAR-LTD induced by LFS (1 Hz, 15min, 900 pulses) to control slices both from neonatal and adolescent animals as shown in Fig. 5E, F. After 45 min, fEPSP slope was 78 ± 2.2% and 81 ± 2.4% in slices from P8-15 and P21-35 mice respectively, moreover 79 ± 2.5% and 82 ± 3.6% in CDK4 inhibitor pretreated slices. These results indicated that cyclinD1-CDK4 are specifically required for mGluR dependent LTD, but do not play a significant role in NMDAR-LTD.

## Discussion

It has been well documented that neurogenesis persists in the adult brain but normally only within discrete regions of the brain containing pluripotent neuronal precursors (e.g. dentate gyrus of adult brains of rodents, primates and humans) [29-34]. Several studies have reported the expression of cyclins and CDKs in terminally differentiated neurons of the dentate gyrus and other brain regions, especially in the context of seizure, transient global ischemia, adrenal steroids, stress or apoptotic events [35-39]. Cell cycle arrest and terminal differentiation of neurons may not be necessarily incompatible with CDK activity but raise the possibility that CDKs and cyclins have physiological impact general effects, such as metabolic regulation [19], basal transcription [20,21] or apoptosis [22,23]. A further function might be implicated in regulating microtubules stability [25] thereby influencing morphoplastic processes.

Our study showed that cell cycle regulatory protein CDK4 had been also located in the cytoplasm and nuclei of postnatal (P10) and even adolescent (P28) neurons in the CA subfields of the hippocampus. The detection of CDK4 in postmitotic neurons suggested a physiological role beyond the regulation of cell cycle G1 checkpoint, because immunoreactivity of it was detected predominantly in perikaryal cytoplasm. A putative function of constitutive cyclin expression in postmitotic neurons has been previously implicated in mechanisms of neuronal and synaptic plasticity [24]. Western blot analyses revealed the presence of CDK4 at the two developmental stages. However, the study was not designed to identify relative changes in the expression levels during the development.

The expression and translocation of G1 cyclins are regulated mostly by PI3K/AKT/mTOR/p70S6K1 signaling [5-8], which is activated by distinct tyrosine kinases and are present in the hippocampus, where they participate in the regulation of synaptic functionality and gene transcription. [40-42]. Our observations of a significant reduction of PPF in slices pretreated with CDK4 inhibitor from neonatal animals (P8-P15) suggested a role of cyclinD1-CDK4 in neurotransmitter release at the early development stage. But there was no significant decrease in PPF when applied to slices from adolescence animals (P21-P35). PTP, another form of short-term synaptic plasticity, was also impaired in CDK4 inhibitor pre-incubated slices from both neonatal and adolescent animals. All these data suggested that cyclinD1-CDK4 mediated short-term synaptic plasticity in hippocampal slices.

Distinct transmitter release properties determine differences in short-term plasticity [43]. This form of synaptic enhancement is caused by a Ca^2+^-dependent increase in release probability and releasable vesicle pool size[44], the residual elevation in pre-synaptic Ca^2+^i acting on one or more molecular targets that appear to be distinct from the secretory trigger responsible for fast exocytosis and phasic release of transmitter to single action potentials. Previous studies suggested that silent synapses are not only exist post-synaptically but also pre-synaptically[45] in developing hippocampal neurons. The available silent synapses can often be rapidly activated, in many instances. These properties enable silent synapses to participate in short-term plasticity[46]. CDK4 inhibitor had dramatic effects on PPF and PTP at the early development stage (P8-15) in our experiments, which is apparent involved in calcium release or the activation of pre-synaptic silent synapse. On the other hand, it was surprised to see such different effect on PPF by CDK4 inhibitor during the development; these findings demonstrating that cyclinD1-CDK4 have a developmental effect change that modulates pre-synaptic function. One hypothesis to consider is that this difference might be due to the developmental change of silent synapse in hippocampal neurons. However, the exactly mechanism interfered with needs further study.

Phosphatidylinositol 3-Kinase(PI3K) is required for the expression but not for the induction or the maintenance of Long-Term Potentiation in the hippocampal CA1 region[41]. However, there is still lack of information concerning the involvement cyclinD1-CDK4 long term synaptic plasticity mechanisms. Our observations found that cyclinD1-CDK4 inhibitor did not affect the induction or the maintenance of LTP both at P8-P15 and at P21-35. Future studies are aimed at determining whether the expression phase of this form of LTP or other forms of LTP are affected.

Two mechanistically distinct forms of LTD coexist in synapses in the CA1 region of the hippocampus [27]. Induction of one form depends on activation of NMDA receptors [27,28,47], while induction of the other depends on activation of metabotropic glutamate receptors [27,48-51]. The locus of mGluR-LTD expression is unclear. Several studies have reported that mGluR-LTD is associated with increases in paired-pulse facilitation [52] and changes in miniature EPSC frequency[27,52], suggesting that the expression of mGluR-LTD is pre-synaptic. In addition, it was reported that the pre-synaptic vesicle release and cycling are altered during mGluR-LTD [53]. However, these findings do not eliminate the possibility of a postsynaptic contribution to mGluR-LTD. For example, it has been shown that mGluR-LTD is dependent on postsynaptic protein synthesis [28]. Recently, Nosyreva and Huber (2005) found that there is a developmental switch in synaptic mechanisms of hippocampal mGluR-dependent LTD [54]. In adolescent animals (P21-P35), mGluR activation induces LTD require protein synthesis, whereas in neonatal animals (P8 -P15), mGluR-LTD is independent of protein synthesis, instead, results in pre-synaptic function. A previous study [42] reported that activation of the PI3K-Akt-mTOR pathway is required for mGlu-LTD, and is known to be required for dendritic protein synthesis, although cannot exclude the possibility of an additional pre-synaptic function. Determination of downstream effectors of PI3K-Akt-mTOR pathway will be necessary for mGluR-LTD to begin to elucidate the identities of the proteins that are synthesized in dendrites during this type of synaptic plasticity.

In this study, we found that mGluR-LTD was impaired in slices from CDK4 inhibitor pretreated slices both from neonatal animals (P8 – P15) and adolescent animals (P21-P35), and the similar effect was present in PPF-LFS-induced LTD. However, the effect of the CDK4 inhibitor at slices from adolescent animals was not as robust as slices from neonatal animals on this form of LTD. This difference may be due to the distinct role of cyclinD1-CDK4 at the two developmental stages during this form of LTD. In conclusion, our findings demonstrated that cyclinD1-CDK4 are required for mGluR-LTD, and the complex may have two different roles during the developmental stage in synaptic mechanisms of hippocampal mGluR-dependent LTD. A more likely possibility is that PI3K is an important regulator of the protein synthesis dependent phase of LTP and regulates neuronal protein synthesis in response to different extracellular stimuli [41,42]. As a downstream effecter of PI3K-Akt-mTOR pathway, at P21-35, cyclinD1-CDK4 may be required for mGluR-dependent LTD through activate the Rb/E2F1 pathway to partly initiate some dendritic protein synthesis that are necessary for the expression of mGluR-LTD, The incomplete blockage of mGluR-LTD, suggest that other signaling pathways may play an other or additional role in mGluR-LTD. Whereas at P8-15, may be due to its pres-synaptic function.

## Conclusion

In this study we observed the expression and sub-cellular localization of cyclinD1-CDK4 during the postnatal development stage, and cyclinD1-CDK4 may mediate STP but not basal synaptic transmission. The induction or the maintenance of long-term potentiation (LTP) in response to a strong tetanus and NMDA receptor-dependent long-term depression (LTD) are normal in hippocampus, whereas mGluR-dependent LTD is significantly inhibited in slices that pre-incubated by CDK4 inhibitor during the postnatal development. These findings demonstrate a function role for cyclinD1-CDK4 in short-term plasticity and in mGluR-dependent synaptic depression in the hippocampus, and suggest that this cyclin-dependent kinase may have different roles during the postnatal development in mice hippocampus area CA1. Future studies aimed at the exactly mechanism interfered with, and the role of activation of cyclinD1-CDK4 in the effect of lead on synaptic plasticity and cell death in hippocampus following development.

## Methods

### Animals

Care of animals and experiments were conducted in accordance with the National Institutes of Health Guideline for the Care and Use of Laboratory Animals. Efforts were made to minimize the number of animals used. All mice were of the C57B6/129 genetic background. Most of the experiments were conducted in the brain slices of mice at postnatal day8 (P8) to 15 (P15), and day21 (P21) to 35 (P35). The number of mice or hippocampal slices used in each individual experiment was indicated as the "*n*" value in the figure legend.

### Tissue preparation and fluorescent microscopy

Neonatal or adolescent mice were perfused with ice-cold saline followed by fixative containing 4% PFA in 0.1 M PBS, pH 7.4. Brains were removed, postfixed in the same solution over night, and subsequently cryoprotected by equilibration with 30% sucrose in PBS. Sections of 30 μm thickness were cut on freezing microtome and collected in 0.1 M TBS, pH 7.4. Antigen unmasking was performed by treating sections with 0.05% trypsin for 30 min at room temperature. After rinsing in PBS, sections were blocked with 5%bovine serum albumin (BSA) in PBS with 0.3% Triton X-100 (PBST + BSA) for 30 min. Afterwards, sections were incubated with anti-CDK4 antibody (1:300, Santa Cruz Biotechnology) overnight at 4°C. Section-bound antibodies were revealed by incubation with biotinylated goat anti-rabbit antiserum (1:1000, 1h, Amersham). All incubation steps were separated by intense washing with TBS. Specificity of labeling was tested by omitting primary antisera. After such incubation, tissue was devoid of immunoreactivity as expected. Images were captured with the digital microscope camera AxioCamHRC running on Axiovision 3.1 Software (both Carl Zeiss Jena) and processed by means of Adobe1 Photoshop1 7.0 (Adobe Systems, Mountain View, CA). Sections were nuclear counter-stained with DAPI.

### Slice preparation

P8-15 and P21-35 mice were decapitated and the brain was quickly removed and immersed in ice-cold artificial cerebrospinal fluid (ACSF) saturated with 95% O_2_/5% CO_2 _and containing 124.0 mM NaCl, 3.0 mM KCl, 1.25 mM KH_2_PO_4_, 26.0 mM NaHCO_3_, 2.0 mM MgSO_4_, 2.5 mM CaCl_2_, and 10.0 mM glucose. Hippocampi were isolated and cut into 400 μm thick transversal slices by a custom-made tissue-slicer. Slices were maintained in ACSF at room temperature for at least 1 h before recording.

### Drug application and electrophysiological recordings

Slices were transferred to a submerged recording chamber, held submerged between two nylon nets, maintained at 30°C, and perfused continuously with ACSF at a rate of 2ml-3ml/min. Field excitatory postsynaptic potentials (fEPSPs) were recorded with glass microelectrodess (1–3 MΩ) filled with ACSF and positioned in stratum radiatum of area CA1. Synaptic responses were reliably evoked every 20sec in the CA1 region of the hippocampus by stimulating Schaffer collaterals with 0.1 ms pulses. A full input-output (I/O) curve was constructed at the beginning of each experiment, the stimulus intensity (0.2 ms duration, 0.033 Hz) selected for baseline measurement was adjusted to elicit about 40% of the maximal slope. Paired pulse facilitation (PPF) was tested by applying two pulses at different intervals (30–300 ms). LTP was induced using high frequency stimulation (HFS; 100 Hz for 1 s), after 20 min of stable baseline recordings. mGluR-LTD was induced by application of 100 μM DHPG (Tocris Cookson) for 5 min or by pairs of stimuli (interstimulus interval, 50 ms) delivered at 1Hz for 15 min (1800pulses; PPF-LFS).

D, L-AP-5 (Sigma) was prepared fresh in ACSF. CDK4 inhibitor (Merck-Calbiochem)(2-Bromo-12,13-dihydro-5H-indolo [2,3-a]pyrrolo [3,4-c]carbazole-5,7(6H)-dione) was dissolved in dimethylsulphoxide (DMSO) at a concentration of 5 μM. DHPG (Tocris Cookson) was prepared as a 1000 × stock in DMSO, used fresh or kept frozen at -20°C. In both cases, the final concentration of DMSO was at most 0.1% v/v after dilution in the recording bath. Slices were pre-incubated with the inhibitors for 20–30 min before DHPG or paired-pulse low-frequency stimulation (PP-LFS). The effects of all of the pharmacological treatments on LTD were evaluated by comparing control and treated slices.

### Data analysis

Normalized data were averaged across experiments and expressed as means ± SEM. The PPF ratio was obtained by dividing the field potential slope from the second pulse (fEPSP2) by that of the first pulse (fEPSP1). One-way ANOVA was performed to determine whether there were significant differences followed by Bonferroni test as post hoc analysis; p < 0.01 indicated difference significance. Origin analysis software (Microcal Software, Northampton, MA) was used in data analysis.

## Authors' contributions

CCL participated in the design of the study, carried out the Western blot, immunohistological and electrophysiological studies, the statistical analyses and the redaction of the manuscript. XML has revised critically and contributed with important intellectual input regarding this manuscript. WHC and SSY have revised critically and contributed with important intellectual input regarding. JTC has made substantial contributions to conception and design of the manuscript. HLW helped to prepare the experimental animals. DYR coordinated the study, provided critical review of the results and manuscript contents, and acquired the funding for the research program. All authors read and approved the final manuscript.
